# Dispersion Polymerization of Polystyrene Particles Using Alcohol as Reaction Medium

**DOI:** 10.1186/s11671-016-1261-8

**Published:** 2016-02-01

**Authors:** Young-Sang Cho, Cheol Hwan Shin, Sujin Han

**Affiliations:** Department of Chemical Engineering and Biotechnology, Korea Polytechnic University, 237, Siheung-si, Gyeonggi-do, 15073 Republic of Korea

**Keywords:** Dispersion polymerization, Polystyrene nanospheres, Colloidal clusters, Self-assembly

## Abstract

In this study, monodisperse polystyrene nanospheres were prepared by dispersion polymerization using alcohol as reaction medium to prepare colloidal clusters of the latex beads. Polyvinylpyrrolidone (PVP) and 2-(methacryloyloxy)ethyltrimethylammonium chloride (MTC) were used as dispersion stabilizer and comonomer, respectively. The particle size could be controlled by adjusting the reactant compositions such as the amount of stabilizer, comonomer, and water in the reactant mixture. The size and monodispersity of the polymeric particles could be also controlled by changing the reaction medium with different alcohols other than ethanol or adjusting the polymerization temperature. The synthesized particles could be self-organized inside water-in-oil emulsion droplets by evaporation-driven self-assembly to produce colloidal clusters of the polymeric nanospheres.

## Background

Over the past decades, the synthetic routes of polymeric latex beads have been developed by various researchers for the applications of biosensors, templates for porous materials, and colloidal crystals [1–3]. Since latex particles have been used in rubber industry during World War II, the synthesis of polymeric particles has attracted much attention in the field of fine chemistry such as ink materials and adhesives [4–7]. In addition, various researches have been conducted for the applications of the polymeric colloids including fiber industry, calibration of electron microscopes, protein recovery, cancer cell removal, cell isolation, and contrast agent for MRI [8–12]. Packing and organization of the particles have been also studied applying self-assembly in the field of colloid science [13].

The fabrication routes of the latex colloids have been developed as various polymerization methods such as emulsion, suspension, or precipitation polymerization schemes. For the control of the size and size distribution of the latex beads, it is necessary to establish suitable synthesis conditions. For this purpose, special techniques have been developed including successive polymerization from seed particles as multi-step polymerization scheme [14]. In addition, morphologies of the polymeric particles could be also controlled by swelling and polymerization of the seeds [15]. Since most researches are focused on emulsion polymerization, robust recipes for the particle synthesis are still necessary to prepare polymeric beads dispersed in organic solvent by proper synthesis method such as dispersion polymerization [16].

In this study, dispersion polymerization of polystyrene nanospheres was performed to control the diameter of the particles by adjusting the reaction parameters such as the amount of stabilizer. The polymerization was carried out at 70 °C using batch-type reactor. As reactants, styrene, AIBN, and PVP were used as monomer, initiator, and stabilizer, respectively, in the reaction medium such as ethanol. The synthesized polystyrene nanospheres were used as building block particles for the self-organization inside emulsion droplets to induce capillary pressure for evaporation-driven self-assembly.

## Methods

### Materials

For the synthesis of polystyrene beads, styrene monomer (99 %), and initiator such as α,α’-azobis(isobutyronitrile) (AIBN: 99 %) were purchased from Daejung Chemicals and Sigma-Aldrich, respectively. 2-(methacryloyloxy)ethyltrimethylammonium chloride (MTC: 72 % aq) was used as cationic comonomer and bought from Aldrich Chemicals. Polyvinylpyrrolidone (PVP K30, Mw = 40,000) was used as stabilizer and purchased from Junsei Chemicals. Ethanol (99.9 %, HPLC grade, Daejung Chemicals) was used as reaction medium.

For the synthesis of colloidal clusters of polystyrene nanospheres, Abil EM 90 (modified polyether-polysiloxane/dimethicone copolyol) was used as emulsifier and purchased from Cosnet. Hexadecane (anhydrous, 99 %) was used as continuous oil phase and bought from Sigma-Aldrich.

### Synthesis of Polystyrene Nanospheres

Dispersion polymerization was performed to synthesize monodisperse polystyrene nanospheres with narrow size distribution. Ethanol as reaction medium containing polyvinylpyrrolidone (PVP) was poured into batch-type reactor where the temperature was maintained as 70 °C. At room temperature, the monomer is not miscible with reaction medium to form turbid mixture, whereas they can be mixed at elevated temperature such as 70 °C to become transparent solution.

Then, suitable amount of styrene and aqueous solution of comonomer (MTC) were added to the reactor during gentle stirring at around 170 to 200 rpm. Before the addition of initiator, nitrogen purging was conducted to remove oxygen from the reaction vessel for 1.5 h. Then, AIBN initiator was added to the reactor and the synthesis continued for 19 h.

### Fabrication of Colloidal Clusters of Polymeric Nanospheres

The polystyrene nanosphere suspension synthesized by dispersion polymerization was washed by centrifugation and redispersion in fresh ethanol to prepare dispersed phase. The emulsion stabilizer, Abil EM90 was dissolved in hexadecane with 3 wt.% as continuous phase. These dispersed and continuous phases were mixed with the volume ratio of 1:3 and emulsified using mechanical homogenizer for 1 min. The resultant emulsion droplets were evaporated by heating at 90 °C for 1 h to induce the capillary pressure for the self-organization of the PS nanospheres. After heating, the clusters of the polystyrene nanospheres were washed with hexane to remove oil phase such as hexadecane, followed by drying for the observation using electron microscope.

### Instruments for Characterizations

The morphologies of the polystyrene particles were observed using field emission scanning electron microscope (FE-SEM; Hitachi-S4700).

## Results and Discussion

**Fig. 1:**
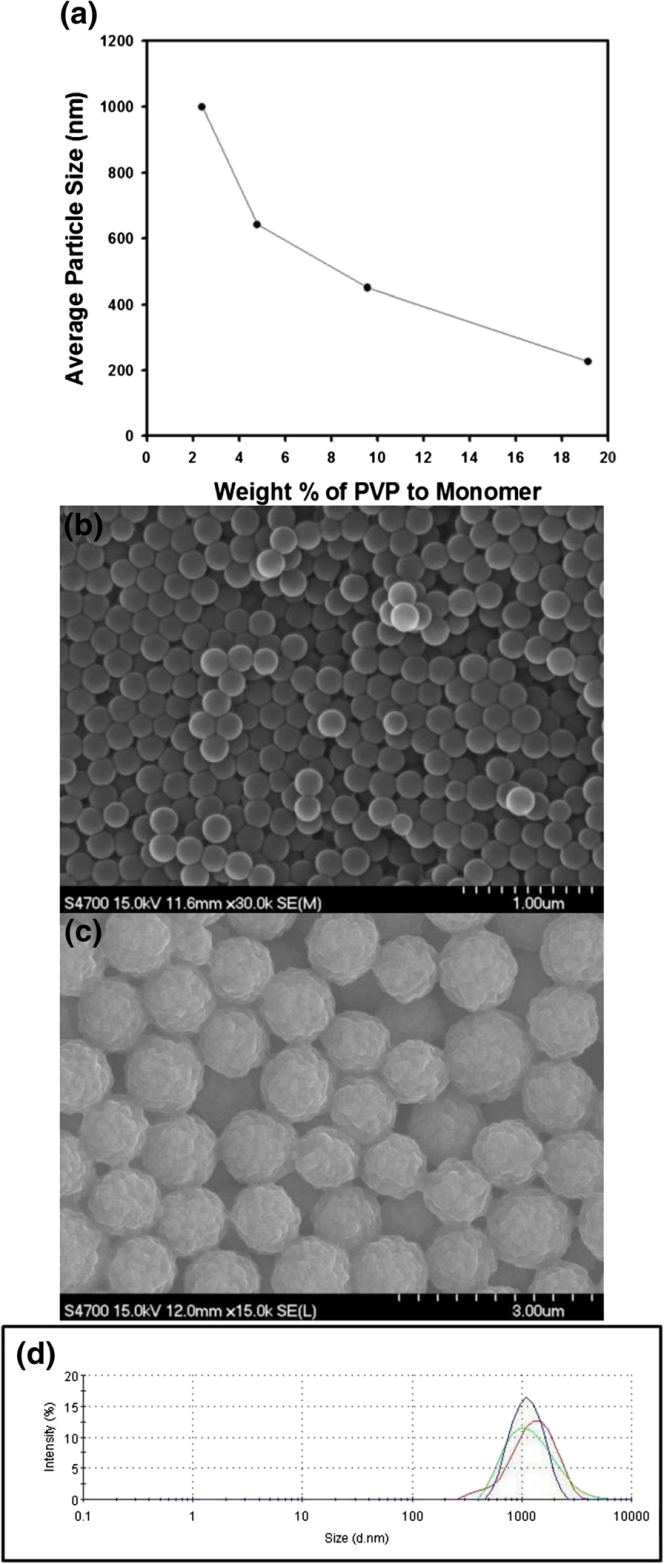
**a** Change of particle diameter as a function of weight percent of PVP to monomer. **b**, **c** Scanning electron microscope image of polystyrene nanospheres synthesized by dispersion polymerization. PVP was used as stabilizer with the amount of (**b**) 8.75 and (**c**) 1.09 wt.%. **d** The size distribution of polystyrene particles shown in Fig. 1c. *Scale bars* indicate 1 and 3 μm for (**b**) and (**c**), respectively. The dispersion polymerization was performed at 70 °C

For the dispersion polymerization of polystyrene particles with narrow size distribution, some polymers such as PVP, polyacrylic acid (PVA), or hydroxypropyl cellulose (HPC) can be adopted as steric stabilizers for preventing the particles from being flocculated [17]. In this study, polar solvent such as ethanol was mainly used as reaction medium for the dispersion polymerization of polystyrene, and the polar molecules such as PVP containing high binding affinity with polar solvents were chosen as stabilizer for the synthesis of monodispersed latex particles. On the contrary, hydrophobic macromolecules such as PDMS can be adopted as stabilizer in dispersion polymerization using nonpolar solvent such as hexane [18]. Figure 1a contains the variation of average particle size as a function of the amount of PVP during dispersion polymerization. Since the role of PVP during particle synthesis is a stabilizer adsorbed on the particle surface, the particle diameter was reduced monotonically with the increasing amount of PVP, as displayed in the graph of Fig. 1a. The average diameter of the polystyrene beads could be controlled from 227 nm to 1 μm by adjusting the amount of stabilizer such as PVP. In this study, the morphologies of the polystyrene beads were observed using scanning electron microscope for the samples prepared with different amount of PVP stabilizer. Figure 1b contains the representative SEM image of the polystyrene nanospheres synthesized by dispersion polymerization, showing that spherical polymeric beads with smooth surfaces could be fabricated. This tendency was maintained as the amount of PVP decreased to 4.375 wt.% with respect to the amount of monomer. However, the surface roughness of the particles increased when the amount of stabilizer was 1.094 wt.%, as displayed in the SEM image of Fig. 1c. When the concentration of the stabilizer decreases, small polystyrene particles generated at the initial stage of polymerization may aggregate to form bigger particles with 1 μm in diameter, resulting in the increase of surface roughness of the particles. Figure 1d contains the size distribution of the polystyrene particles as shown in Fig. 1c, indicating that the average diameter around about 1 μm was maintained during the repeated measurements. Thus, the rapid flocculation of the particle suspension was not observed for the sample shown in Fig. 1c.

**Fig. 2:**
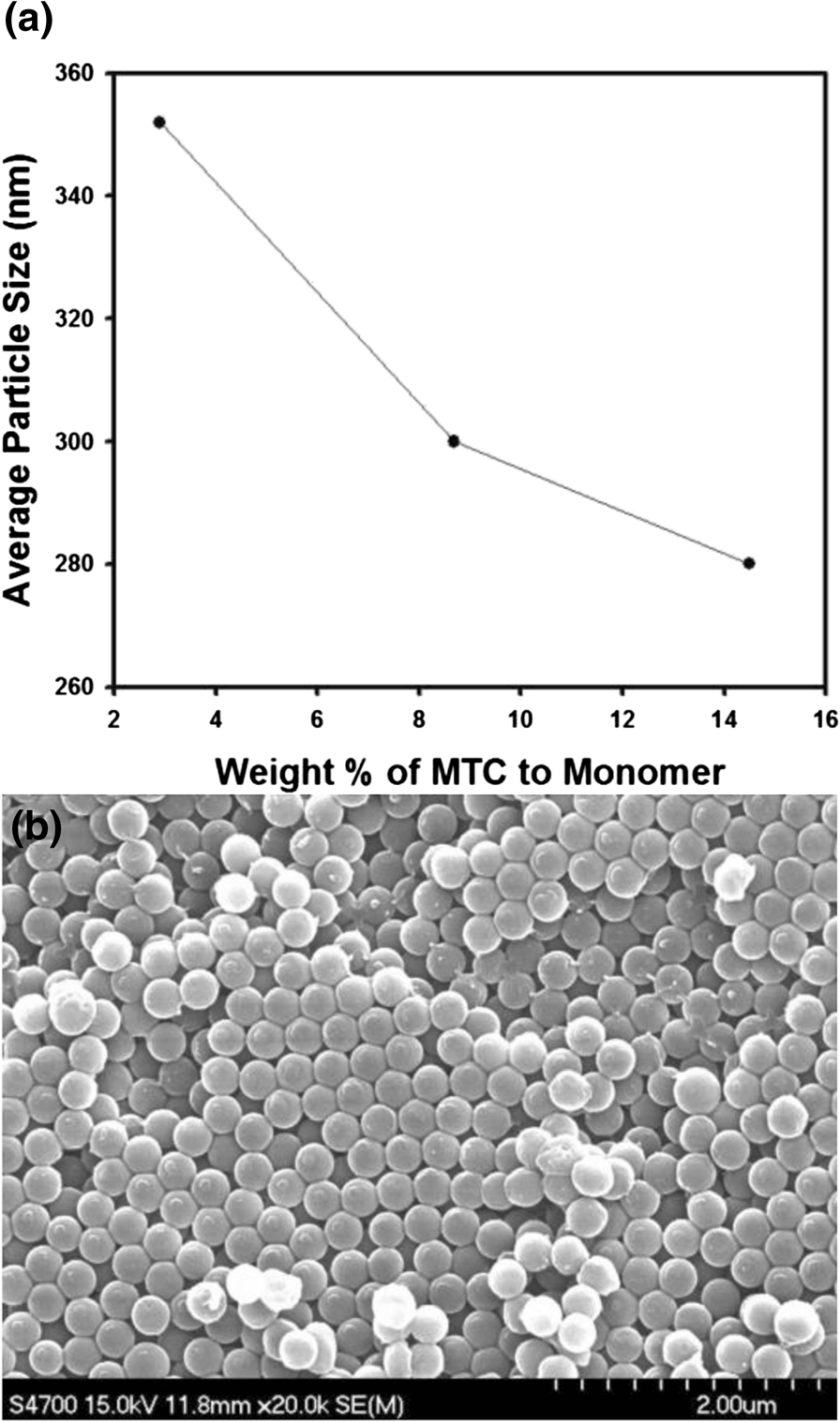
**a** The change of average diameter of polystyrene nanospheres as a function of the amount of MTC. **b** Scanning electron microscope image of polystyrene nanospheres synthesized by dispersion polymerization using 2.9 wt.% of MTC as comonomer. *Scale bars* indicate 2 μm

In addition to PVP, MTC also plays an important role for the stabilization of polystyrene particles since surface electrical charge due to the amine groups of MTC can be created on the particles. Besides the amount of PVP as stabilizer, the concentration of MTC was also adjusted to control the particle diameter during dispersion polymerization. The results are plotted as the graph in Fig. 2a, which contains the trend of the reduction of particle size with increasing concentration of MTC. Since the surface of polystyrene nanospheres can be decorated with amine functional groups derived from MTC, the surface electric charge can be increased by increasing the amount of the comonomer, resulting in the size reduction of the particles, as displayed in the graph of Fig. 2a. Figure 2b displays the scanning electron microscope image of monodisperse polystyrene nanospheres with 352 nm in diameter, which was synthesized using 2.9 wt.% of MTC with respect to the amount of styrene monomer. As displayed in the SEM image, the uniform spherical particles could be fabricated and the size could be controlled from 280 to 352 nm by adjusting the amount of MTC.

**Fig. 3:**
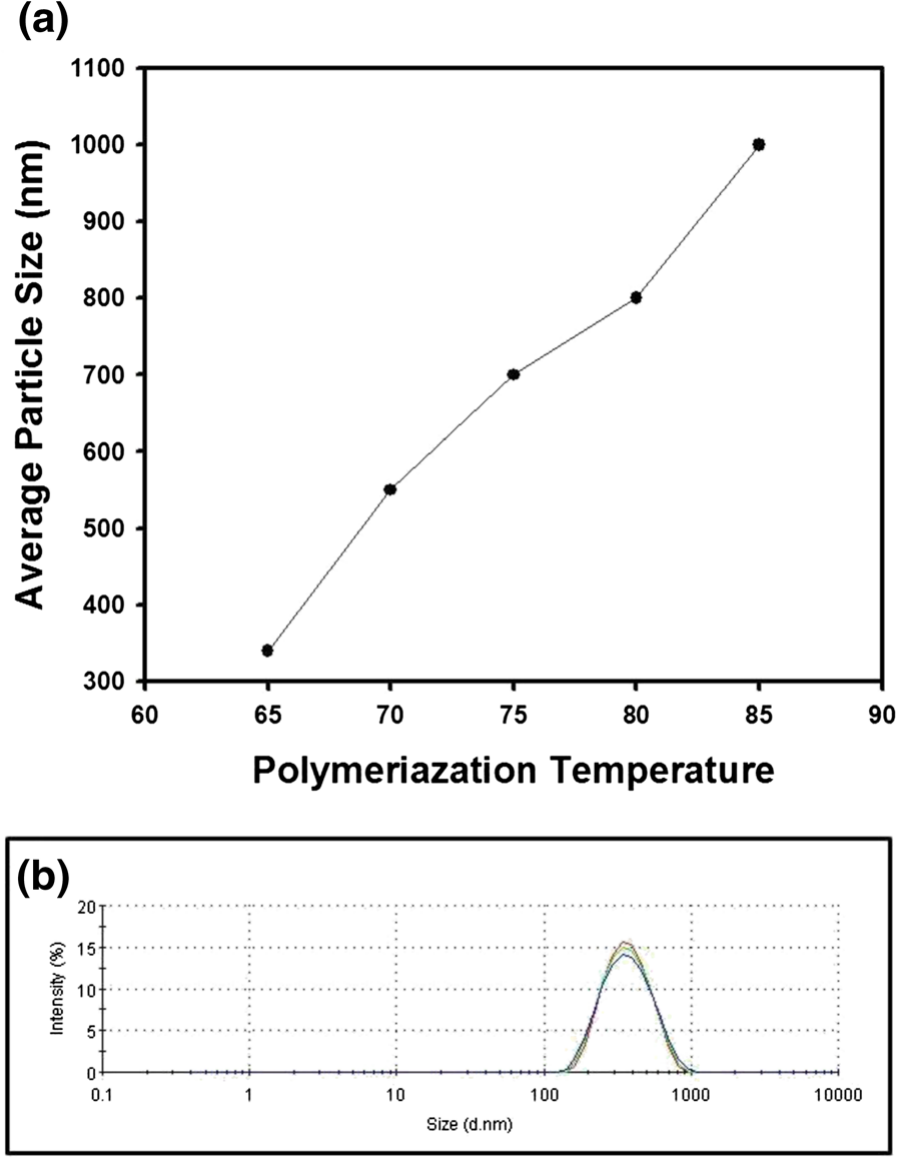
**a** The change of particle diameter as a function of polymerization temperature. **b** The size distribution of the polystyrene nanospheres synthesized at 65 °C

Thus far, the polymerization temperature was fixed as 70 °C to synthesize monodisperse polystyrene nanospheres in ethanol as dispersion medium. The temperature was also changed from 65 to 85 °C to control the particle size of the polymeric beads as contained in the graph of Fig. 3a. As the polymerization temperature increased, the particle diameter became larger, as displayed in the graph of Fig. 3a, showing the trend of monotonic increase. Since the dissociation rate of the initiator such as AIBN increases as temperature increases, the radical concentration in monomer droplets will be increased, resulting in the enlargement of the particle diameter as a function of polymerization temperature. On the contrary, reverse trend was observed for the case of emulsion polymerization since the number of nuclei increases as the polymerization temperature increases, causing the decrease of the particle diameter [13]. Since the initiators molecules are dissolved in monomer droplets and reaction medium during dispersion and emulsion polymerization, respectively, the temperature dependences of the particle size were reversely observed for the two kinds of the synthesis methods. Figure 3b contains the size distribution of the polystyrene nanospheres synthesized at 65 °C, indicating that monomodal particles could be synthesized and the dispersion stability can be maintained since the distribution curves coincide well each other for repeated measurements.

**Fig. 4:**
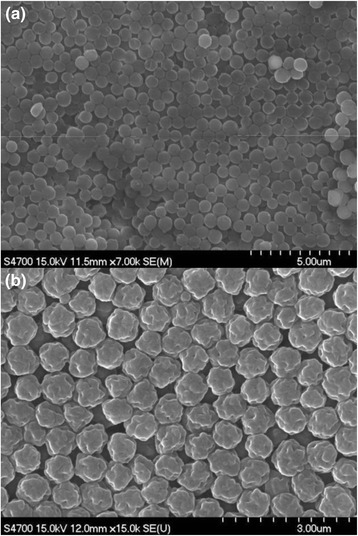
Scanning electron microscope image of polystyrene nanospheres synthesized by dispersion polymerization using ethanol as reaction medium with (**a**) 70 and (**b**) 75 °C as polymerization temperature. *Scale bars* indicate 5 and 3 μm for (**a**) and (**b**), respectively

Figure 4a and b display the scanning electron microscope image of polystyrene nanospheres synthesized at 70 and 75 °C, respectively, using ethanol as reaction medium. The abrupt change of particle morphologies was observed between these two reaction conditions, indicating that the surface roughness depended on the polymerization temperature as well as the amount of stabilizer. The solvency of the stabilizer such as PVP can be affected by the polymerization temperature, and poor solvency of PVP may induce the precipitation of bigger particles from the reaction medium, resulting in the formation of the odd-shaped particles shown in the microscope image of Fig. 4b. Similar morphologies of the particles have been observed during the dispersion polymerization of cross-linked particles depending on the solvency of the solvent. Poor solvency led to the formation of odd-shaped particles due to the formation of shorter chain of polymers and their precipitates [19].

**Fig. 5:**
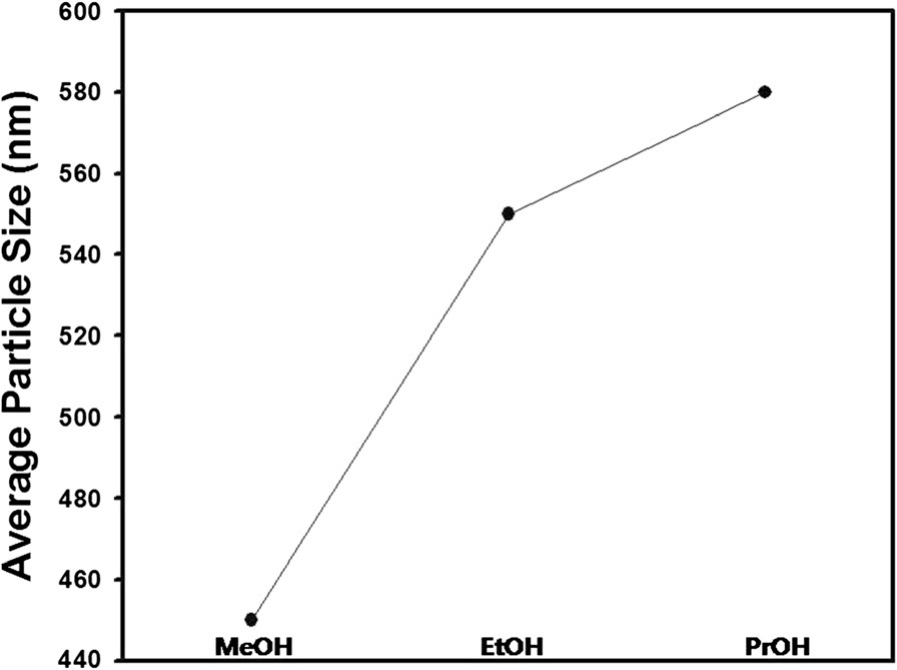
Change of particle diameter with different alcohols as reaction medium. The polymerization temperature was fixed as 70 °C

In this study, other alcohols such as methanol and 2-propanol were also adopted as reaction medium for dispersion polymerization of polystyrene latex particles. Figure 5 contains the change of the average particle size obtained with different reaction medium such as methanol, ethanol, and propanol. When heavy alcohols were used, the diameter of the synthesized polystyrene nanospheres increased under the same fabrication conditions. Since the solvent with high dielectric constant effectively screen the attractive or repulsive forces, methanol can be used as efficient medium to screen the van der Waals force between particles [20]. Since the Hamaker constant *A* is proportional to the difference of the dielectric constant between the particles and dispersion medium, reaction medium such as methanol having relatively high dielectric constant has the tendency to increase the van der Waals force, according to the following equation [21].

1 $$ A=\frac{3}{4}kT{\left(\frac{\varepsilon_A-{\varepsilon}_B}{\varepsilon_A+{\varepsilon}_B}\right)}^2+\frac{3h\nu }{16\sqrt{2}}\frac{{\left({n_A}^2-{n_B}^2\right)}^2}{{\left({n_A}^2+{n_B}^2\right)}^{1/2}} $$

Here, *ε*_*A*_ and *ε*_*B*_ denote the dielectric constant of particle and dispersion medium, respectively, whereas *n*_*A*_ and *n*_*B*_ stand for the refractive index. In Eq. (1), *k* and *h* represent Boltzmann and Plank constant, respectively. However, the attractive van der Waals force can be screened effectively due to the increase of the dielectric constant of the surrounding solvent such as methanol, indicating that the steric stabilization becomes more important. The potential energy *V* between the particles can be represented as the summation of van der Waals force, electrostatic repulsive force, and steric force *V*_*s*_, as shown in the following equation [21].

2 $$ V=-\frac{1}{12}\frac{A}{Ha}+{\varPsi}_0{e}^{-\kappa H}+{V}_s $$

Here, *H* and *a* stand for the separation distance between the particles and the particle radius, respectively. *Ψ*_*0*_ and *1/κ* denote the surface electric potential and the electric double layer thickness, respectively. Since the *1/κ* is proportional to the square root of the dielectric constant, the maximum range of electrostatic force increases with decreasing value of dielectric constant. However, the particle size was increased with decreasing value of the dielectric constant of solvent, indicating that steric force has more strong contribution of the particle diameter. In Eq. (2), the steric force *V*_*s*_ due to PVP molecules adsorbed on the particle surface also plays an important role for the stabilization of the polymeric particles and the diameter of the particles. The solution state of PVP can be changed in heavy alcohols since the steric force of the stabilizing agent may be decreased in such alcohols due to the change of the radius of gyration. Thus, the particle growth could be observed by adopting the alcohols with high molecular weight, as shown in the graph of Fig. 5.

**Fig. 6:**
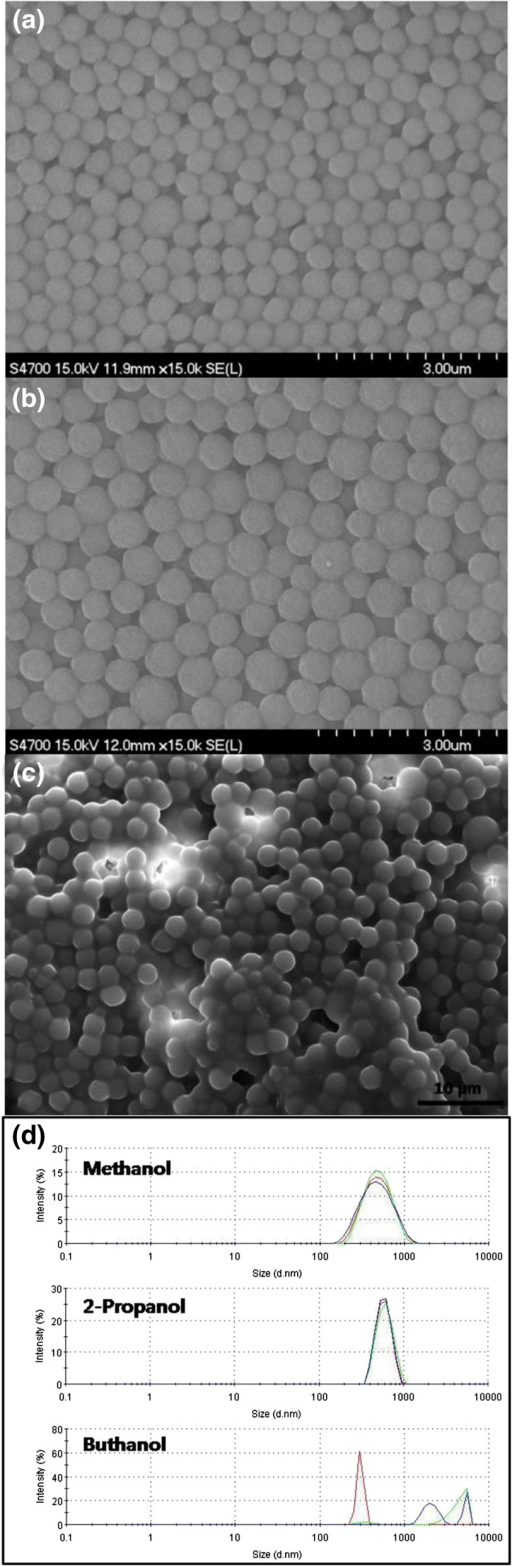
Scanning electron microscope image of polystyrene particles synthesized by dispersion polymerization using (**a**) methanol, (**b**) 2-propanol, and (**c**) butanol as reaction medium. *Scale bars* indicate 3 μm for (**a**) and (**b**). For Fig. 6c, scale bar is 10 μm. **d** Size distribution of particle dispersion shown in Fig. 6 **a**–**c**

Figure 6a and b display the scanning electron microscope image of the polystyrene nanospheres synthesized using methanol and 2-propanol as reaction medium. In addition to the average size of the particles, the polystyrene nanospheres became less monodisperse when methanol or 2-propanol was used as reaction medium. When butanol was used as reaction medium, the polystyrene particles became micron-size and very polydisperse as contained in the scanning electron microscope image of the polymeric particles as shown in Fig. 6c. Dynamic light scattering was applied to measure the size distribution of the polystyrene nanospheres shown in the electron microscope image of Fig. 6a–c, indicating that the particle suspension synthesized using butanol as reaction medium is unstable since size enlargement was observed during repeated measurements. On the contrary, the size and distribution of the polymeric suspension were maintained uniformly for the polystyrene nanospheres synthesized using methanol and 2-propanol, implying that the steric effect of PVP as stabilizer is valid in small alcohols as dispersion medium. However, PVP seemed to be less effective in butanol medium, possibly due to the decrease of radius of gyration of the grafting polymers in heavy alcohols.

**Fig. 7:**
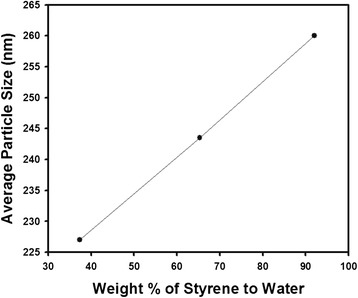
Change of particle diameter as a function of the weight percent of styrene with respect to the amount of water. The polymerization temperature was fixed as 70 °C, and ethanol was used as reaction medium

In this article, the reactant composition was adjusted by changing the amount of water to study the variation of the size of polystyrene nanospheres. Figure 7 contains the change of particle size as a function of the amount of styrene with respect to the amount of water. As contained in the graph of Fig. 7, the size reduction was observed as the increasing amount of water, possibly due to the increase of dielectric constant of dispersion medium. Also important to note is that the surface electric charge due to amine groups derived from MTC would increase with increasing amount of water in the reaction medium, affecting the size reduction of the polystyrene nanospheres. Since the miscibility of styrene decreases with increasing amount of water, the addition of water may cause the gradual change from dispersion polymerization to emulsion polymerization, resulting in the size reduction of the polymeric particles. Usually, emulsion polymerization can be applied to prepare submicron-sized particles whereas dispersion polymerization can be adopted for the synthesis of larger particles with 2 to 12 μm in diameter [22]. If homogeneous solution cannot be maintained due to excess amount of water, hetero-phase polymerization may be started out from the monomer source of immiscible droplets and smaller particles can be favored from the monomer-swollen micelles.

**Fig. 8:**
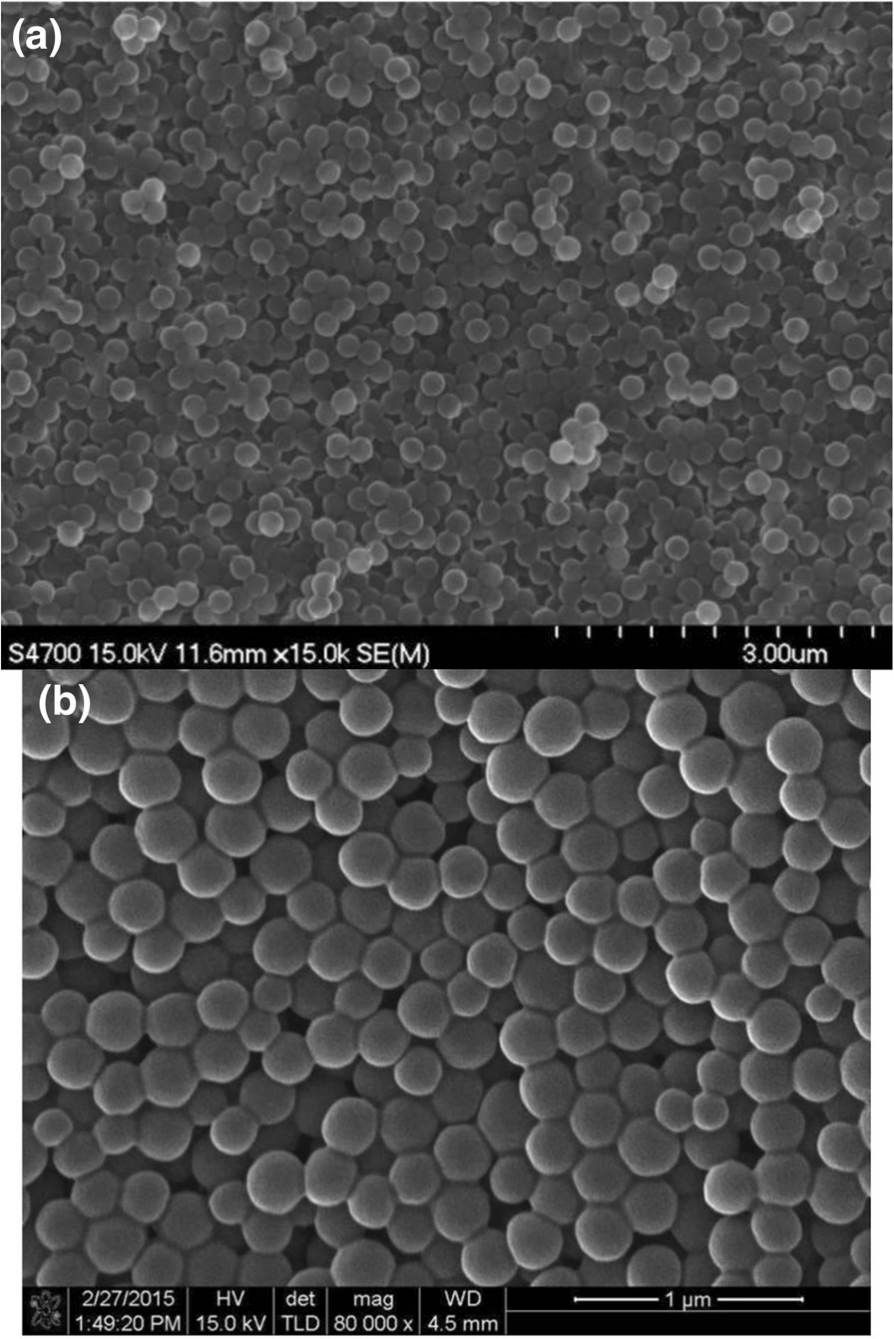
Scanning electron microscope image of polystyrene nanospheres synthesized by dispersion polymerization using ethanol as reaction medium at 70 °C as polymerization temperature. The weight percent of styrene to water was maintained as (**a**) 62.3 and (**b**) 95.03, respectively. *Scale bars* indicate 3 and 1 μm for (**a**) and (**b**), respectively

Figure 8a and b contains the scanning electron microscope image of the polystyrene nanospheres synthesized using ethanol as reaction medium with the weight percent of styrene to water as 62.3 and 95.03, respectively. As contained in the SEM images, the size monodispersity can be enhanced by increasing the amount of water in reaction medium.

**Fig. 9:**
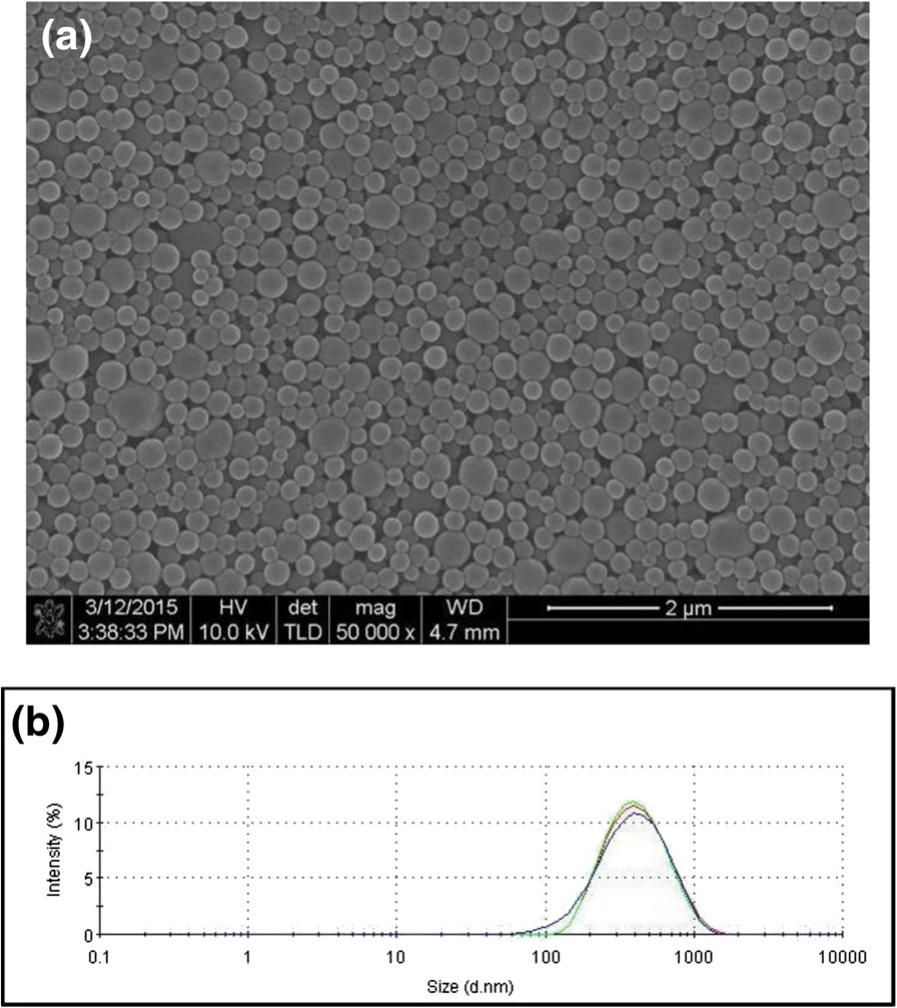
**a** Scanning electron microscope image and (**b**) size distribution of polystyrene nanospheres synthesized by dispersion polymerization using ethanol and PVA as reaction medium and stabilizer, respectively. The polymerization was performed at 70 °C. *Scale bar* indicates 2 μm

Besides PVP, polyvinyl alcohol (PVA) was used as stabilizer during dispersion polymerization. Figure 9a contains the scanning electron microscope image of the resultant polymeric beads synthesized using ethanol as reaction medium at 70 °C. Since the solubility of PVA in ethanol is not as good as PVP, the size distribution of the polystyrene nanospheres was relatively broader than that of polymeric beads stabilized with PVP, as displayed in the SEM image of Fig. 9a. Figure 9b contains the size distribution of the polymeric dispersion displayed in Fig. 9a, showing broader distribution of the particles compared to the polystyrene nanospheres synthesized using PVP as stabilizer.

**Fig. 10:**
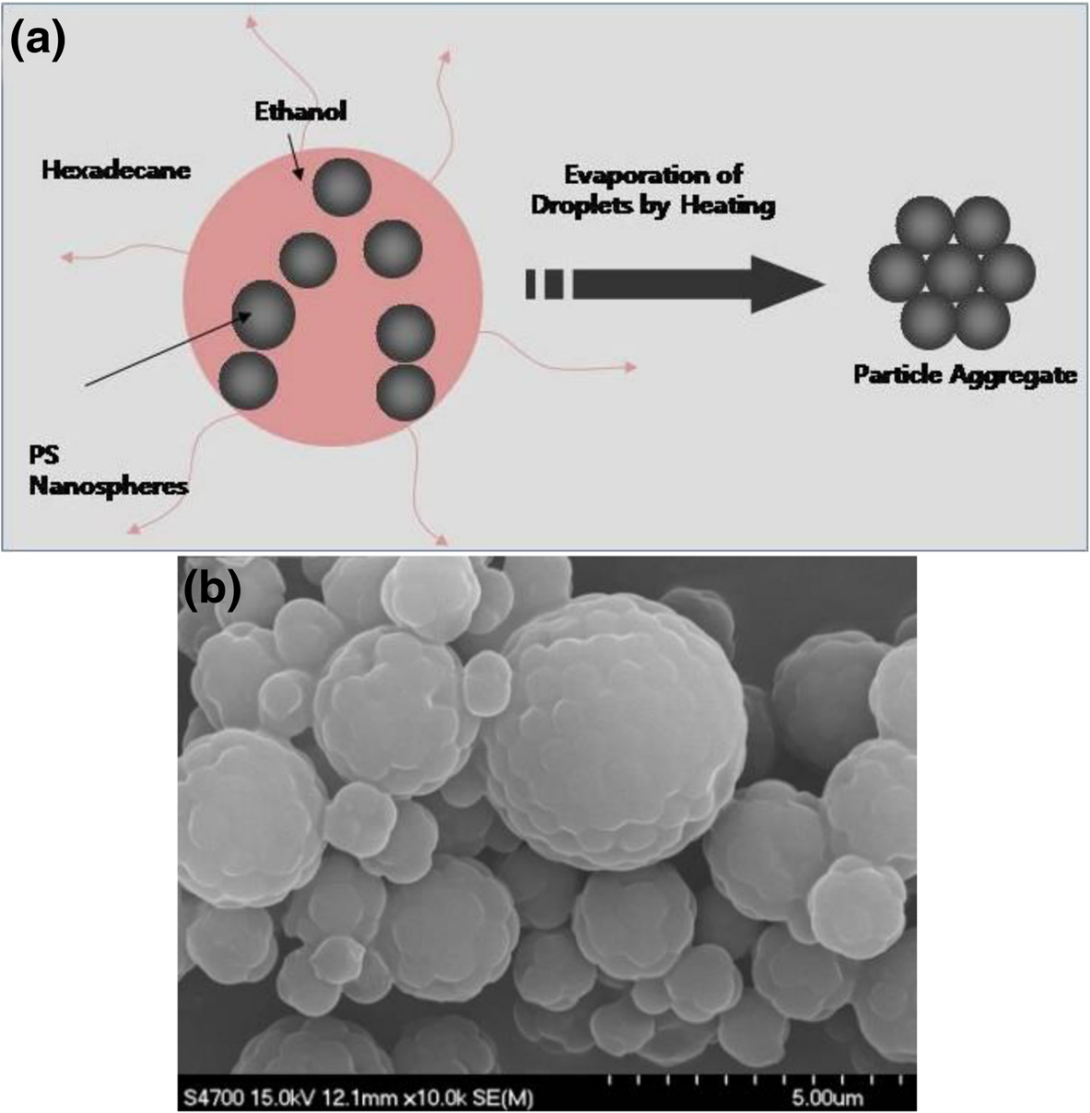
**a** Schematic figure for the fabrication of colloidal aggregates of polystyrene nanospheres. **b** Scanning electron microscope image of the aggregates of the polystyrene nanospheres synthesized by dispersion polymerization. *Scale bars* indicates 5 μm

Figure 10a displays the schematic figure for the formation of supraparticles composed of polystyrene nanospheres. After the polymeric latex beads suspended in ethanol they were prepared by dispersion polymerization, they were emulsified with oil phase such as hexadecane by mechanical homogenization. The volatile solvent inside droplets was evaporated by heating, causing inward capillary force to assemble the polymeric particles into supra-aggregates. Figure 10b contains the scanning electron microscope image of the supraparticles assembled with the polystyrene nanospheres with 642 nm in diameter as building block particles. The polymeric beads were synthesized using 2.188 wt.% of PVP as stabilizer during the dispersion polymerization at 70 °C. For the assembly of the particles inside emulsions, the droplets were removed by heating at 90 °C for 1 h, resulting in the formation of the clusters shown in the SEM image of Fig. 10b. Due to the heating process, the polymeric beads were partially fused between the particles due to sintering effect.

## Conclusions

Monodisperse polystyrene nanospheres were synthesized by dispersion polymerization in alcohol as reaction medium, and the particle size was controlled by adjusting the reaction parameters such as polymerization temperature and reactant compositions. The effect of the amount of the stabilizer such as PVP, comonomer, and water were studied systemically to control the average diameter of the polymeric particles. The size range of the polystyrene latex could be adjusted from 200 nm to 1 μm. Besides the particle diameter, monodispersity was also affected when different types of reaction medium or stabilizing agent were used during dispersion polymerization. The synthesized particles were adopted as building blocks for the fabrication of clusters or supraparticles by evaporation-driven self-assembly using water-in-hexadecane emulsions as confining geometries.
